# An annotated aerial imagery dataset for automated detection of harbour seals in Svalbard, Norway

**DOI:** 10.1038/s41597-026-07297-x

**Published:** 2026-05-20

**Authors:** Zoé Lemoine, Puneet Sharma, Kit M. Kovacs, Christian Lydersen, Marie-Anne Blanchet

**Affiliations:** 1 Norwegian Polar Institute (Norsk Polarinstitutt), FRAM Centre, Tromsø, Norway; 2 University of La Rochelle, La Rochelle, France; 3 UiT The Arctic University of Norway, Tromsø, Norway

**Keywords:** Conservation biology, Climate-change ecology, Boreal ecology, Marine biology, Image processing

## Abstract

Arctic marine ecosystems are undergoing significant changes due to rapid climate warming, affecting species abundances and distributions. Harbour seals (Phoca vitulina), the most widely distributed pinniped in the world, are expanding their range northward in Arctic waters. Monitoring their ongoing distributional shift using traditional survey methods is logistically challenging. In particular, detecting and manually counting seals from aerial imagery is labour-intensive and costly. Integrating object detection algorithms into the analytical phase of surveys can greatly reduce post-processing time and costs. Here, we present 495 annotated images from the High Arctic Svalbard Archipelago with 7,085 harbour seal annotations highlighted as oriented bounding boxes. The dataset includes challenging background conditions with well-camouflaged and sparsely distributed animals, providing a valuable resource that complements existing datasets. While Svalbard is of particular ecological relevance as the fastest-warming region on the planet with advanced climate-driven range shifts, the detection methodology enabled by this dataset is applicable across the species’ full geographic range. It supports the development of automated object detection models for ecological monitoring and population studies worldwide.

## Background & Summary

Global biodiversity is continuously reshaped by complex interactions between organisms and their environments, contributing to ecosystem resilience and function over time^1,2^. However, anthropogenic pressures and climate change are causing rapid and unprecedented disruptions to these natural dynamics, leading to shifts in species distributions, abundance, and community compositions^3,4^. Monitoring these changes is essential to anticipate long-term ecological consequences^5^.

The Arctic is one of the most vulnerable regions to climate warming. It is currently experiencing temperature increases at rates 2–5 times the global average^6^. The Svalbard Archipelago, located in the Northeast Atlantic Arctic is being particularly heavily affected by rising temperatures, diminishing sea ice cover, and changing oceanographic conditions^7–10^. These abiotic changes are driving significant alterations in marine species distributions, including the northward expansion of temperate taxa into previously ice-covered areas^11–14^.

The harbour seal (*Phoca vitulina*) is the most widely distributed pinniped species, inhabiting coastal regions across the North Atlantic and North Pacific^15^. Traditionally found in temperate areas, it is not an ice-associated species, but it has maintained a small population along the west coast of Prins Karls Forland, Svalbard^15–17^. Over the last decade, it is very likely that this population has been increasing and it has definitely expanded its range. This population’s dynamics offer valuable insights into ecological shifts in Arctic marine environments under climate change. However, monitoring marine mammals in remote and vast regions remains logistically challenging^18^. Monitoring wildlife abundance and distribution typically relies on methods such as direct counts, mark–recapture, acoustic surveys, and aerial or satellite imageries^19,20^. In the Arctic, these approaches are constrained by vast, inaccessible areas, harsh and rapidly changing weather, limited seasonal windows, and high operational costs^18^. For harbour seals, this task is particularly challenging as individuals spend much of their time at sea, but their predictable haul-out behaviour on land provides an opportunity for monitoring using aerial imagery. Moreover, manual image analysis, which is still widely used, requires visually counting of each individual, a labour-intensive and expensive process^21,22^. Such factors make it difficult to conduct frequent, large-scale surveys, leading to sparse datasets and potential gaps in long-term monitoring.

**Fig. 1:**
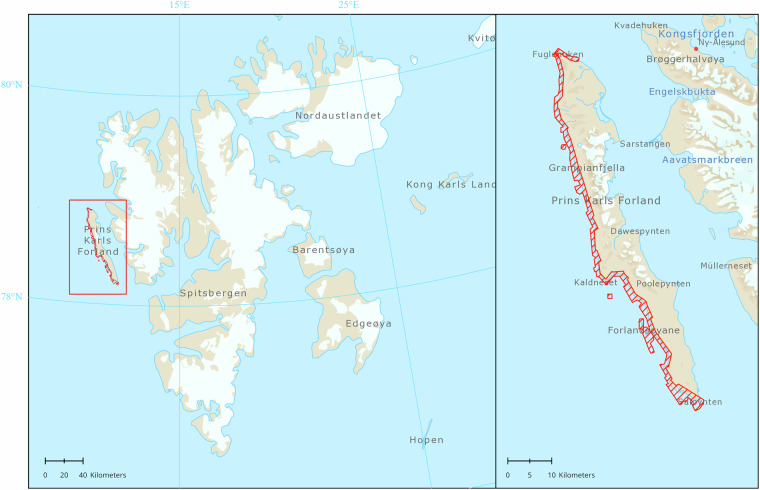
The map on the left shows the location of Prins Karls Forland within the Svalbard Archipelago. The map on the right provides a detailed view of the study area: Prins Karls Forland. The hatched areas indicate the zones photographed during the 2009 and 2010 survey campaigns. Map data: NP_Basiskart_Svalbard_WMS, Norwegian Polar Institute.

We developed the SealDetectionDataset^23^, a curated collection of aerial image tiles annotated with bounding boxes identifying individual seals to help alleviate traditional survey challenges. The dataset^23^ was constructed from high-resolution aerial images obtained during three survey flights over the coastlines of Prins Karls Forland, Svalbard, on 1 August 2009, and 1 August and 19 August 2010 (Fig. 1). These images were originally collected for an abundance estimate of this population^24^. Here we have worked with this dataset to support machine learning applications for automated detection. All harbour seals visible in the aerial images were manually annotated with bounding boxes. The resulting dataset^23^ consists of 495 annotated image tiles (800 × 800 pixels), with 7,085 individual seal annotations provided in Pascal VOC format. The dataset^23^ captures seals in realistic and often challenging conditions, including heterogeneous coastal habitats and backgrounds where individuals are well camouflaged and sparsely distributed.

The SealDetectionDataset^23^ provides a valuable resource for developing automated detection approaches that can be applied across diverse geographic regions where harbour seals occur. It can complement other seal datasets, by providing images from a different geographic region with complex backgrounds and light conditions. For machine learning applications, combining datasets from diverse environments increases variability in training data, which improves the robustness and generalizability of object detection models^25–27^. These carefully annotated data are made publicly available to advance research in ecological monitoring and to support the application of machine learning approaches for improving wildlife survey efficiency and accuracy in challenging environments.

## Methods

### Study area and image acquisition

The study area is located along the western coast of Prins Karls Forland, part of the Svalbard Archipelago, Norway. This region hosts the world’s northernmost population of harbour seals^28^. Aerial surveys were conducted during three flights on 1 August 2009, 1 August and 19 August 2010. Flights took place in the afternoon during low tide and under favorable weather conditions, which are prime haul-out conditions for harbour seals^17^. A Twin Commander 690 C fixed-wing aircraft equipped with a Microsoft Vexcel Ultracam XP camera was used to collect high-resolution images. The camera, mounted on a gyrostabilized platform with a 100.5 mm focal length lens, captured images every two seconds at a flight altitude of approximately 670 meters and a flight speed of 300 km/h. Each image covered approximately 0.31 km² with 60% overlap between consecutive images, enabling stereoscopic analysis. To visualize the study area, maps were generated using ArcGIS Pro (version 3.4.0, Esri).

### Creating image tiles

**Fig. 2:**
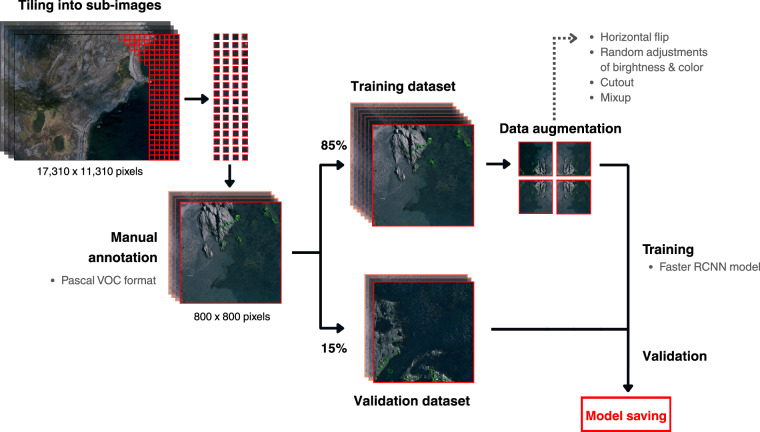
Pipeline for dataset preparation from aerial imagery and training of the seal detection model.

From the original high-resolution aerial images acquired during the 2009 and 2010 survey campaigns, smaller sub-images (image tiles) of 800 × 800 pixels were created with a 10% overlap to facilitate annotation and analysis (Fig. 2). For the 2009 campaign, which consisted of a single survey day that obtained 975 large aerial TIFF images, additional manual cropping of the large images was performed to increase the number of sub-images. In 2010 the survey campaign included two survey days, capturing 995 images from 1 August and 980 images from 19 August, resulting in approximately twice as many aerial images compared to 2009. All images and sub-images were stored in TIFF format.

### Creating labelled images

A subset of the generated image tiles containing visible harbour seals was manually annotated using the open-source software LabelImg. For the 2009 campaign, 304 image tiles were annotated, while 87 and 104 tiles were annotated for the 1 August and 19 August 2010 campaigns, respectively. Bounding boxes were drawn as tightly as possible around each seal to accurately delineate the animal’s location. Annotations were saved in the Pascal VOC format, with bounding box coordinates stored in individual XML files corresponding to each image chip.

### Data usage

This dataset^23^ is well-suited for training and evaluating object detection models in machine learning, especially deep learning. Its high-resolution images and precise annotations enable the development of automated tools for detecting the harbour seals. Spatial information, such as coordinates, is not included in this tiled dataset. Although other seal species are present in the area, only harbour seals were detected and annotated in this dataset. While the dataset^23^ has not yet been tested in other geographic regions, under different environmental conditions, or in the presence of other seal species; exploring its applicability in new areas is one of its intended uses, and it may be complemented with additional annotations. It can support ecological monitoring, population assessments, and serve as a benchmark for remote sensing applications.

## Data Records

The SealDetectionDataset^23^ is publicly available at the Norwegian Polar Institute repository using the DOI link: 10.21334/NPOLAR.2025.C4E51ECE. It consists of aerial image tiles and corresponding annotation files organized by survey date. For each survey date, the image tiles are stored in TIFF format with filenames following the pattern “seal_ < date > _ < tile_id > .tif”, where the name encodes the date of acquisition and a unique tile identifier. The annotation files are provided in Pascal VOC XML format, where each XML file corresponds to one image tile and contains bounding box coordinates precisely marking the location of each visible harbour seal, labelled as “seal.” These annotation files follow the same naming convention as the image tiles, with the extension “.xml” instead of “.tif”.

## Technical Validation

### Annotation consistency

All harbour seals in the selected image tiles were manually annotated using the LabelImg tool. Annotators followed a consistent procedure to draw bounding boxes tightly around each visible seal, ensuring spatial accuracy. Seal identification was based on a combination of visual characteristics, including body shape, size, coloration, and contextual information such as grouping behaviour and proximity to shoreline features. The images used in this dataset were previously analysed in an abundance estimation study by Merkel *et al*.^24^, where each image was examined by two trained observers and cross-validated. Only images confirmed to contain at least one seal were retained. Furthermore, the present annotations were performed by a trained observer under direct supervision by an experienced seal researcher, using established visual criteria. The annotations were saved in Pascal VOC XML format. A verification step was carried out by visually inspecting each annotated image to ensure that object boundaries were correctly placed and that no objects were missed.

### Use of Data for an object detection model

To validate the quality and utility of the SealDetectionDataset^23^, we trained and evaluated a deep learning object detection model using the annotated images (Fig. 2). We used a Faster R-CNN architecture^29^ with a ResNet-50 backbone and a Feature Pyramid Network (FPN), a widely used object detection model. Two models were trained: the first using only the 2009 data, and the second using a combined dataset from both 2009 and 2010, to assess the impact of data diversity on detection performance. For training, data augmentation techniques such as horizontal flip, random contrast and brightness, cutout and mixup were applied to increase variability in the input images^30,31^. Model performance was evaluated using standard object detection metrics. The second model achieved better performance: 73% precision, 80% recall, and a mean IoU (Intersection over Union) of 76%, demonstrating the benefit of incorporating data from multiple surveys. Although the second model outperformed the first, some false negatives remained, particularly for the few seals in water. These omissions were relatively infrequent, and the model succeeded in reliably locating seal presence in large aerial images, significantly reducing the time needed for manual inspection. These results indicate that, while visual verification of detections is necessary, the dataset is sound and effective for training automated seal detection models from aerial images. The use of more advanced deep learning models would be essential for improving the detection performance. The proposed dataset^23^ is suitable to meet the needs of current and advanced object detection models.

## Data Availability

The SealDetectionDataset^23^ is publicly available at the Norwegian Polar Institute repository under 10.21334/NPOLAR.2025.C4E51ECE. It contains aerial image tiles in TIFF format with corresponding annotation files in Pascal VOC XML format, organized by survey date.

## Code availability

No custom code was developed for processing this dataset beyond standard preprocessing and annotation tools. The annotations were created using LabelImg, and standard deep learning frameworks (e.g., PyTorch) were used for model training.
